# Classification of cognitive impairment in older adults based on brain functional state measurement data via hierarchical clustering analysis

**DOI:** 10.3389/fnagi.2023.1198481

**Published:** 2023-12-15

**Authors:** Yangxiaoxue Liu, Na Wang, Xinling Su, Tianshu Zhao, Jiali Zhang, Yuhan Geng, Ning Wang, Ming Zhou, Gongzi Zhang, Liping Huang

**Affiliations:** ^1^Medical School of Chinese PLA, Beijing, China; ^2^Department of Rehabilitation Medicine, The First Medical Center, Chinese PLA General Hospital, Beijing, China; ^3^School of Sport Medicine and Rehabilitation, Beijing Sport University, Beijing, China

**Keywords:** cognitive impairment, brain functional state measurement, aging, electroencephalogram, hierarchical clustering analysis

## Abstract

**Introduction:**

Cognitive impairment (CI) is a common degenerative condition in the older population. However, the current methods for assessing CI are not based on brain functional state, which leads to delayed diagnosis, limiting the initiatives towards achieving early interventions.

**Methods:**

A total of one hundred and forty-nine community-dwelling older adults were recruited. Montreal Cognitive Assessment (MoCA) and Mini-Mental State Exam (MMSE) were used to screen for CI, while brain functional was assessed by brain functional state measurement (BFSM) based on electroencephalogram. Bain functional state indicators associated with CI were selected by lasso and logistic regression models (LRM). We then classified the CI participants based on the selected variables using hierarchical clustering analysis.

**Results:**

Eighty-one participants with CI detected by MoCA were divided into five groups. Cluster 1 had relatively lower brain functional states. Cluster 2 had highest mental task-switching index (MTSi, 13.7 ± 3.4), Cluster 3 had the highest sensory threshold index (STi, 29.9 ± 7.7), Cluster 4 had high mental fatigue index (MFi) and cluster 5 had the highest mental refractory period index (MRPi), and external apprehension index (EAi) (21.6 ± 4.4, 35.4 ± 17.7, respectively). Thirty-three participants with CI detected by MMSE were divided into 3 categories. Cluster 1 had the highest introspective intensity index (IIi, 63.4 ± 20.0), anxiety tendency index (ATi, 67.2 ± 13.6), emotional resistance index (ERi, 50.2 ± 11.9), and hypoxia index (Hi, 41.8 ± 8.3). Cluster 2 had the highest implicit cognitive threshold index (ICTi, 87.2 ± 12.7), and cognitive efficiency index (CEi, 213.8 ± 72.0). Cluster 3 had higher STi. The classifications both showed well intra-group consistency and inter-group variability.

**Conclusion:**

In our study, BFSM-based classification can be used to identify clinically and brain-functionally relevant CI subtypes, by which clinicians can perform personalized early rehabilitation.

## Introduction

The aging population is an important global demographic characteristic. Currently, China is witnessing an accelerated aging process. Data from the UN Population Division have suggested that the percentage of the population aged 65 years 11.5 per cent in 2019 to 16.9 per cent in 2030 in China (Nations U, 2019). Cognitive impairment (CI) is a common degenerative condition related to aging (Gauthier et al., 2006). According to WHO reports, more than 50 million people had dementia in 2019 and by 2030 the number is expected to reach 82 million (The Lancet, 2018; World Health Organization, 2022). CI is the leading cause of disability and mortality in older adults (Gonzales et al., 2022; World Health Organization, 2017). Dementia leads to increased costs for governments, communities and families, and reduce economic productivity. By 2030, it is estimated that the cost of caring for people with dementia worldwide will be as high as $2 trillion (World Health Organization, 2022).

Obtaining an accurate diagnostic tool for early CI is crucial in preventing the onset of CI and treating lately diagnosed CI. Presently, there are two approaches accepted for assessing CI including the Montreal Cognitive Assessment (MoCA) (Nasreddine et al., 2005) and Mini-Mental State Exam (MMSE) (Folstein et al., 1975). Previous studies have demonstrated that MoCA has higher sensitivity and diagnostic accuracy than MMSE (Dong et al., 2012; Trzepacz et al., 2015; Ciesielska et al., 2016), while MMSE is superior to MoCA in discriminating CI in individuals with lower education levels (Zhang et al., 2021). However, these relatively brief scales with subjective assessment indices cannot accurately identify and classify patients, thus failing to guide individualized treatment.

CI is reported to be associated with decreased brain function (Culig et al., 2022). Identifying and classifying patients with CI according to the brain function state can effectively guide the implementation of rehabilitation, such as repetitive transcranial magnetic stimulation (rTMS) (Gomes-Osman et al., 2018). Electroencephalogram (EEG) is a non-invasive tool that continuously monitors brain activity in frontal, temporal, parietal and occipital areas and reflects functional changes in brain regions (Jorge et al., 2014; Rubinos et al., 2020). EEG-based brain network appears to be a reliable tool that can be used to identify neuropathological and cognitive alterations in people with neurodegenerative brain disorders (Frantzidis et al., 2014). EEG serves as a non-invasive biological tool for evaluating CI, and an abnormal quantitative EEG signal is listed as a supportive biomarker for dementia with Lewy bodies (McKeith et al., 2017; Martin et al., 2022). However, EEG data is a non-stationary random signal with strong background noise, causing difficulty in analysis and low recognition rate (Shang et al., 2023; Wang et al., 2023).

Brain functional state measurement (BFSM) is a new technology to comprehensively analyze the relative power spectrum of EEG (Sai et al., 2018; Alturki et al., 2020). Based on wavelet calculation, BFSM could be used to obtain the characteristic quantitative indicators of the brain state contained in the brain wave. Over the past decade, studies have confirmed the important role played by BFSM in monitoring concentration, relaxation, anxiety, alertness, and intelligence levels in individuals (Ren et al., 2011; Luo et al., 2022).

The purpose of the present investigation was to explore the relationship between brain function and cognitive decline. Accordingly, we recruited a large sample of participants and conducted the cognitive assessment with MoCA, MMSE, and prefrontal-BFSM. Using unsupervised hierarchical clustering analysis, we aimed to classify the patients with CI according to their brain state, test the intra-group consistency and inter-group variability, and then identify the characteristics of each group so as to obtain relevant data that could guide therapy.

## Materials and methods

### Participants

A total of 149 community-dwelling older people (aged from 60–99 years) were recruited in the Fall Clinic of Chinese PLA General Hospital. The inclusion criteria were as follows: (a) age ≥ 60 years; (b) primary school education and above, could understand the content of the scale; and (c) voluntary participation and informed consent. Exclusion criteria were the following: (a) those with a history of central nervous system disease or neuromuscular disease; (b) severe illness that prevents completion of the scale or questionnaire assessment; (c) severe interference during the measurement of brain functional state; (d) severe alcohol or substance abuse/dependence; and (e) severe visual and auditory impairment. A total of 134 participants (≥60 years old) were included in the final analysis. Fifteen participants were excluded, for refusing to completing MMSE or MoCA (*n* = 3), failing to complete the visuospatial executive function test due to hand tremor (*n* = 2), failing to complete MoCA due to poor visualization (*n* = 4), incomplete academic information (*n* = 2), and incorrect date of birth (*n* = 2). This study was approved by the Ethics Committee of the General Hospital of the People’s Liberation Army (S2022-475).

### Index of BFSM

#### Theoretical basis

The EEG signals recorded on the scalp’s surface are a composite reflection of the electric fields generated by the activity of neurons and synapses in the brain (Buzsáki et al., 2012). Activities of the nervous system causes changes in brain waves to form different manifestations of brain function. Potential changes on the scalp surface could reflect functional brain information, including cortical and subcortical neuronal information (Seeber et al., 2019).

#### Testing procedure

The index of the brain’s functional state was recorded by the portable HXD-I multi-functional combined monitor. EEG electrode placement was performed according to the international scalp electrode positioning 10/20 system (Jurcak et al., 2007). Non-invasive EEG electrodes were placed 2 cm above the center point of the forehead and eyebrows (FZ), above the brow arches bilaterally (left FP_1_, right FP_2_), and reference electrodes were placed in the ear lobes bilaterally (left A_1_, right A_2_). BFSM was started after the patient rested for 2 min, and the effective EEG signal was continuously recorded for 6 min. EEG data collected during this process included eye-closing, second eye-opening, and maintaining concentration under real-time voice prompts specifying system tasks (a detailed method is shown in Supplementary materials).

### Cognitive function test

The cognitive state of participants was evaluated using MoCA and MMSE. Higher scores in MoCA and MMSE indicated better cognitive performances. Scores of >25 and below for the MoCA and scores of >26 and below for the MMSE were denoted to differentiate CI or dementia from normal. We did not limit the minimum scores for the MMSE and MOCA in the inclusion criteria.

### Data analysis

A least absolute shrinkage and selection operator (LASSO) regression model implemented in the ‘glmnet’ package for R was established to select the optimal predictors of CI. This method can minimize the prediction error by penalizing the absolute magnitude of the regression model coefficients, causing the regression coefficients of weak variables to shrink toward zero and only retaining variables with non-zero coefficients. The indicators for CI were further screened out by univariate logistic regression analysis based on *p*-value, in which the cutoff was 0.1.

To analyze all types of participants with CI, the selected variables were used for hierarchical clustering analysis, which is a statistical method to organize a series of samples into unique, mutually exclusive groups by clustering some similar characteristics of data points. The smallest distance showed the highest degree of relationship, indicating that those objects were more likely to cluster in a group. The main characteristics of different clusters were analyzed.

In order to test the intra-group consistency and inter-group variability, analysis of similarities (ANOSIM) and analysis of variance (ANOVA) was performed. ANOSIM was performed on “Euclidean” distance measures for the hierarchical clusters by using the R package “vegan.” The statistical significance of the observed R was assessed by 10^4^ permutations. The significantly larger difference between groups than that within groups suggested the rationality of clustering of CI participants.

## Results

### Overview

A total of 134 participants (≥60 years old) were included in the final analysis. Participants with cerebrovascular disease, Alzheimer’s disease, and education at or below the primary school level were excluded. According to MoCA, 81 participants with a mean age of 77.9 ± 10.8 years, 35 (43.2%) of whom were female, were evaluated as suffering from CI. According to MMSE, 33 participants with a mean age of 80.0 ± 11.4 years, 13 (39.4%) of whom were female, were evaluated as suffering from CI. Of the 134 participants, MoCA scores ranged from 2 to 30 and MMSE scores ranged from 8 to 30. Twenty-eight participants were diagnosed with CI by both the MoCA and MMSE. Baseline characteristics of all participants are summarized in Table 1.

**Table 1:** Baseline characteristics of all participants.

Name	MoCA			MMSE		
	No CI (*N* = 53)	CI (*N* = 81)	*p*-value	NoCI (*N* = 101)	CI (*N* = 33)	*p*-value
Sex (Female)	35 (66%)	35 (43.2%)	0.016	57 (56.4%)	13 (39.4%)	0.133
Age (Mean ± SD)	68.7 ± 6.9	77.9 ± 10.8	<0.001	72.3 ± 9.4	80.0 ± 11.4	<0.001
Education	/	/	/	/	/	/
Middle school	6 (11.3%)	14 (17.3%)	0.617	14 (13.9%)	6 (18.2%)	0.833
College	22 (41.5%)	33 (40.7%)	/	42 (41.6%)	13 (39.4%)	/
University	25 (47.2%)	34 (42%)	/	45 (44.6%)	14 (42.4%)	/
Visuospatial executive (Mean ± SD)	4.5 ± 0.6	3.1 ± 1.2	<0.001	/	/	/
Naming (Mean ± SD)	2.9 ± 0.3	2.7 ± 0.7	0.014	/	/	/
Attention (Mean ± SD)	5.8 ± 0.4	5.2 ± 1.1	<0.001	/	/	/
Language (Mean ± SD)	2.2 ± 0.7	1.8 ± 0.9	0.004	/	/	/
Abstraction (Mean ± SD)	1.9 ± 0.4	1.3 ± 0.7	<0.001	/	/	/
Delayed recall (Mean ± SD)	3.9 ± 0.8	1.7 ± 1.6	<0.001	2.1 ± 1.0	0.5 ± 0.7	<0.001
Orientation (Mean ± SD)	5.9 ± 0.2	5.4 ± 1.1	<0.001	9.9 ± 0.4	8.5 ± 2.8	0.008
Registration (Mean ± SD)	/	/	/	3.0 ± 0.2	2.7 ± 0.8	0.063
Attention and calculation (Mean ± SD)	/	/	/	4.8 ± 0.5	3.2 ± 1.5	<0.001
Language and praxis (Mean ± SD)	/	/	/	8.9 ± 0.4	8.2 ± 1.1	0.005
MoCA (Mean ± SD)	27.3 ± 1.3	21.3 ± 4.2	<0.001	/	/	/
MMSE (Mean ± SD)	/	/	/	28.5 ± 1.2	23.1 ± 4.6	<0.001

### LASSO and univariable analysis

The LASSO model was used to analyze CI-related indicators in BFSM. The brain function state score was the independent variable, while CI was the dependent variable. In the LASSO regression model with MoCA as the diagnostic criterion for CI, five variables with non-zero coefficients were retained as potential predictors, including mental refractory period index (MRPi), external apprehension index (EAi), sensory threshold index (STi), mental fatigue index (MFi), mental task-switching index (MTSi) (Supplementary Figure S1). In the LASSO regression model with diagnostic criteria of MMSE, 10 indicators had non-zero coefficient estimates (Supplementary Figure S1). The AUC of the two LASSO regression were 0.72 (MoCA) and 0.75 (MMSE), respectively (Supplementary Figure S2). The variables selected by LASSO regression were further screened out by univariable regression (Table 2; Supplementary Figure S3), after which three variables [brain metabolism rate index (BMRi, MRPi, MFi)] were excluded from the hierarchical clustering analysis of CI identified by MMSE.

**Table 2:** Univariate regression further screened the selected variables of LASSO regression.

Name	MoCA			MMSE			
	No CI (*N* = 53)	CI (*N* = 81)	*p*-value	No CI (*N* = 101)	CI (*N* = 33)	*p*-value	
Age (Mean ± SD)	68.7 ± 6.9	77.9 ± 10.8	<0.001	72.3 ± 9.4	80.0 ± 11.4	<0.001	
Sex (Female)	35 (66%)	35 (43.2%)	0.010	57 (56.4%)	13 (39.4%)	0.092	
BMRi (Mean ± SD)	/	/	/	162.0 ± 107.6	186.6 ± 114.3	0.263	
MRPi (Mean ± SD)	4.2 ± 4.8	7.3 ± 7.7	0.014*	5.5 ± 6.2	7.8 ± 8.6	0.103	
CEi (Mean ± SD)	/	/	/	209.4 ± 69.0	180.0 ± 67.0	0.038*	
Hi (Mean ± SD)	/	/	/	30.8 ± 10.1	34.9 ± 7.8	0.037*	
ERi (Mean ± SD)	/	/	/	19.9 ± 13.2	26.6 ± 17.6	0.025*	
ATi (Mean ± SD)	/	/	/	18.8 ± 18.0	26.2 ± 24.8	0.071*	
EAi (Mean ± SD)	27.9 ± 11.3	23.0 ± 14.3	0.039*	/	/	/	
STi (Mean ± SD)	16.9 ± 11.5	11.5 ± 9.8	0.006*	14.6 ± 10.9	10.7 ± 10.1	0.078*	
IIi (Mean ± SD)	/	/	/	28.6 ± 13.1	34.8 ± 20.2	0.050*	
ICTi (Mean ± SD)	/	/	/	73.6 ± 20.4	63.9 ± 25.1	0.030*	
MFi (Mean ± SD)	45.3 ± 29.5	59.7 ± 33.4	0.013*	52.6 ± 31.8	58.3 ± 34.9	0.376	
MTSi (Mean ± SD)	9.9 ± 7.3	8.0 ± 5.0	0.086*	/	/	/	

### Hierarchical clustering analysis

**Figure 1:**
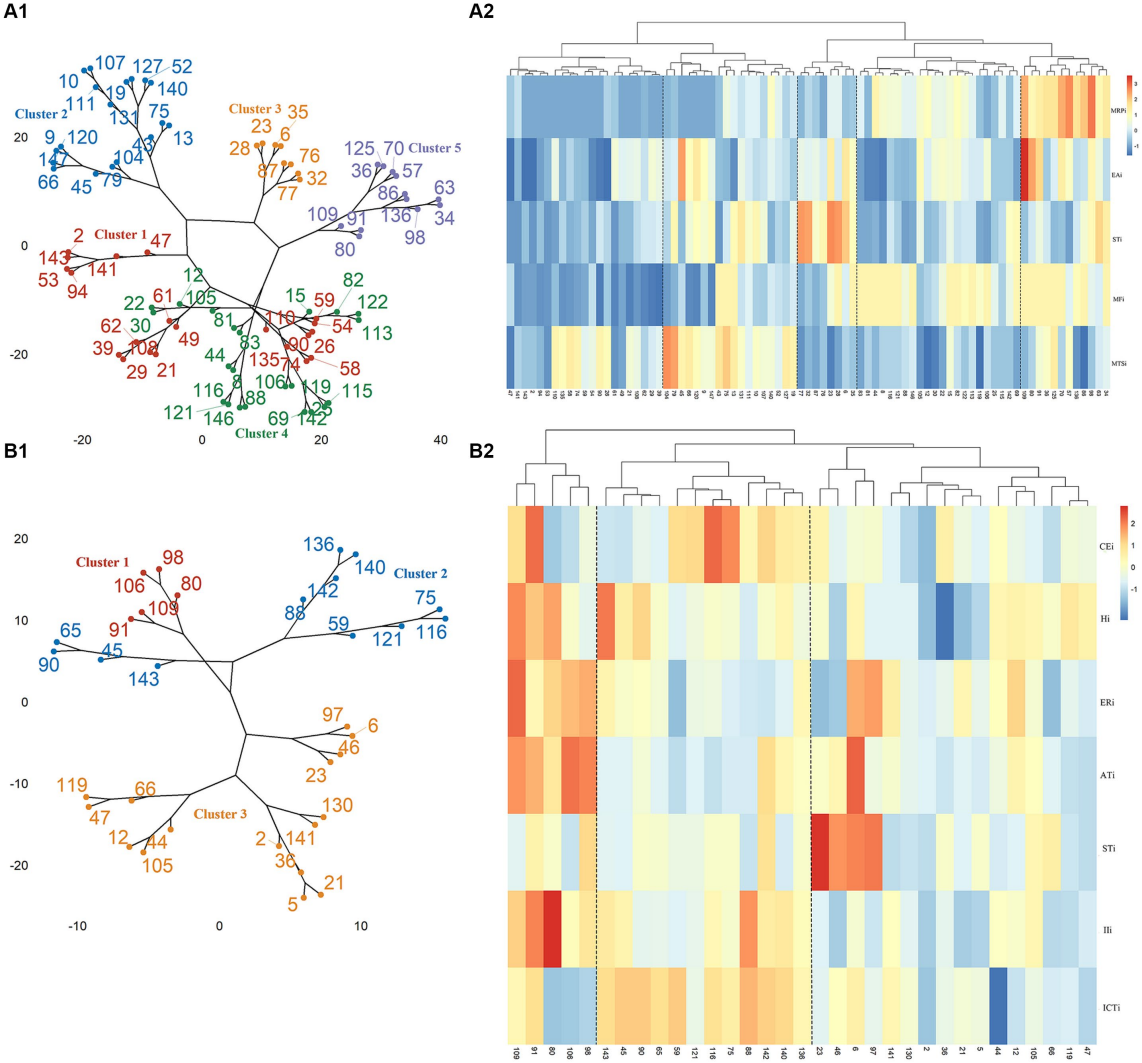
Cluster analysis and correlation heat map of participants. **(A1)** Cluster analysis of participants diagnosed with CI by MoCA. **(A2)** Correlation heat map of MoCA scores and CI-related indicator scores in BMFS. **(B1)** Cluster analysis of participants diagnosed with CI by MMSE. **(B2)** Correlation heat map of MMSE scores and CI-related indicator scores in BMFS.

**Figure 2:**
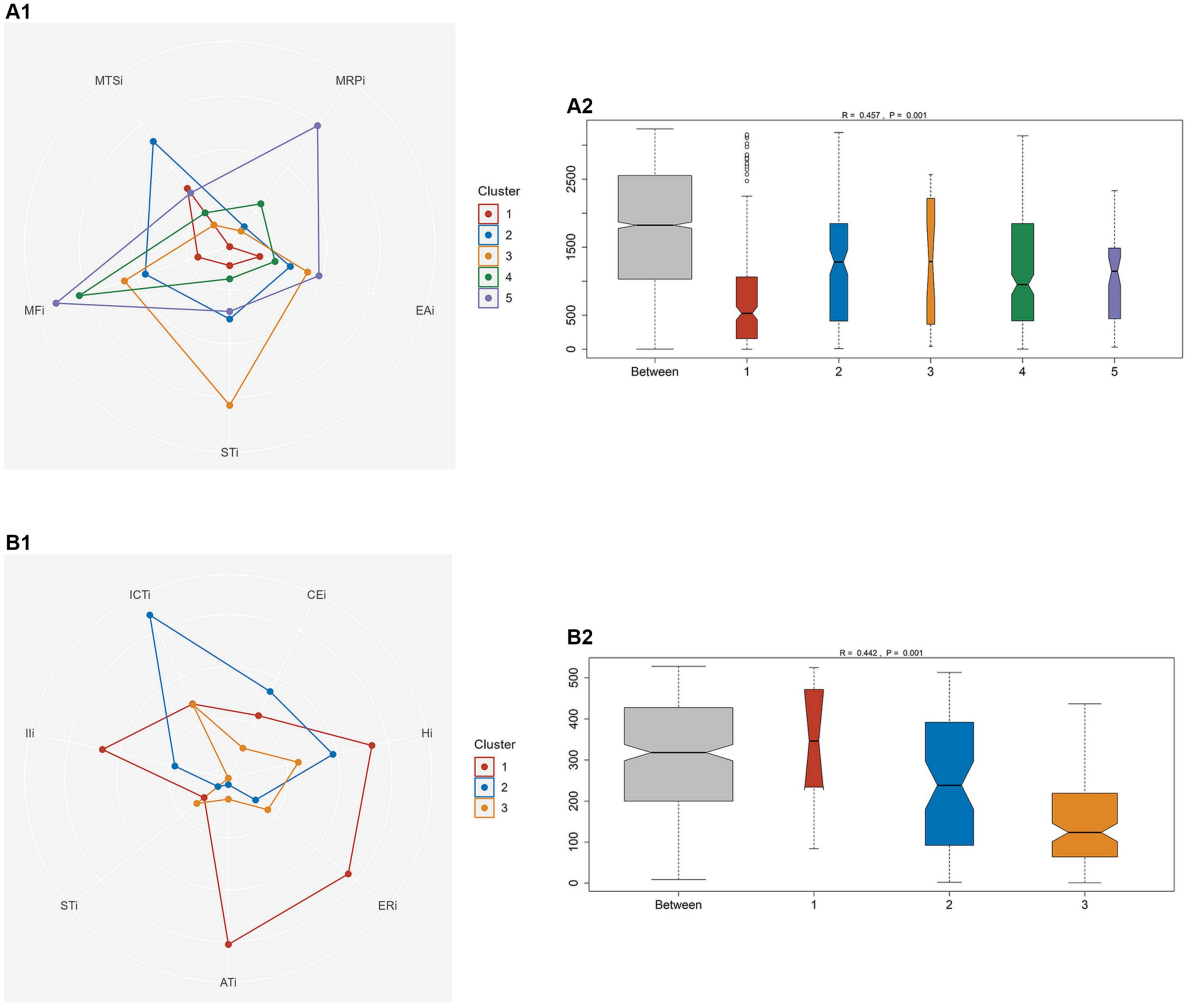
One-way analysis of similarity (ANOSIM) between different clusters to test the intra-group consistency and inter-group variability. **(A)** Participants with CI diagnosed by MoCA. **(B)** Participants with CI diagnosed by MMSE.

BFSM indicators were used for cluster analysis of participants diagnosed with CI by MoCA (*n* = 81) and MMSE (*n* = 33), respectively. The optimal number of clusters was determined by voting method (Supplementary Figure S4). Relational grades of different samples depend upon the distance in the dendrogram, where the shortest distance shows the highest similarity. The participants with CI diagnosed by MoCA were divided into 5 clusters (Figure 1). According to variance analysis and radar plot (Table 3; Figure 2), CI-related indicators differed in different clusters (*p* < 0.001), and five classifications showed well inter-group variability. Participants in cluster 1 had lower indexes than other clusters, except MTSi; participants in cluster 2 had the highest MTSi (13.7 ± 3.4) and lower MFi; those cluster 3 had the highest STi (29.9 ± 7.7) and lower MTSi and MRPi; those in cluster 4 had higher MFi, and those in cluster 5 had the highest MFi, MRPi, and EAi (90.8 ± 15.7, 21.6 ± 4.4, 35.4 ± 17.7), and lower STi. Cluster 1 participants were characterized by sensory impairment, Cluster 2 participants had a better functional state. Cluster 3 participants with low reaction speed, and Cluster 4 participants were characterized by low reaction speed and sensory impairment. Cluster 5 participants were characterized by reduced brain functional reserve.

**Table 3:** Variance analysis of CI-related indicators in different clusters.

Name	MoCA						MMSE			
	Cluster 1 (*N* = 21)	Cluster 2 (*N* = 18)	Cluster 3 (*N* = 8)	Cluster 4 (*N* = 22)	Cluster 5 (*N* = 12)	*p*	Cluster 1 (*N* = 5)	Cluster 2 (*N* = 12)	Cluster 3 (*N* = 16)	*p*
MRPi (Mean ± SD)	1.4 ± 3.0	4.7 ± 4.4	4.0 ± 3.4	8.5 ± 5.2	21.6 ± 4.4	<0.001	/	/	/	/
EAi (Mean ± SD)	14.2 ± 11.3	25.2 ± 12.6	31.4 ± 8.7	19.7 ± 11.2	35.4 ± 17.7	<0.001	/	/	/	/
STi (Mean ± SD)	5.1 ± 4.2	14.6 ± 8.1	29.9 ± 7.7	7.5 ± 6.3	13.2 ± 9.1	<0.001	11.0 ± 8.4	8.4 ± 5.1	12.4 ± 13.2	0.607
MFi (Mean ± SD)	27.5 ± 20.6	50.9 ± 27.9	60.2 ± 29.8	80.4 ± 26.2	90.8 ± 15.7	<0.001	/	/	/	/
MTSi (Mean ± SD)	8.0 ± 4.5	13.7 ± 3.4	3.6 ± 1.7	5.1 ± 3.0	7.5 ± 4.5	<0.001	/	/	/	/
CEi (Mean ± SD)	/	/	/	/	/	/	187.6 ± 97.9	213.8 ± 72.0	152.2 ± 38.0	0.047
Hi (Mean ± SD)	/	/	/	/	/	/	41.8 ± 8.3	36.4 ± 6.2	31.6 ± 7.4	0.022
ERi (Mean ± SD)	/	/	/	/	/	/	50.2 ± 11.9	20.1 ± 10.1	24.1 ± 18.0	0.002
ATi (Mean ± SD)	/	/	/	/	/	/	67.2 ± 13.6	16.2 ± 15.6	20.8 ± 20.4	<0.001
IIi (Mean ± SD)	/	/	/	/	/	/	63.4 ± 20.0	39.7 ± 14.9	22.1 ± 11.7	<0.001
ICTi (Mean ± SD)	/	/	/	/	/	/	50.8 ± 28.2	87.2 ± 12.7	50.5 ± 18.2	<0.001

According to MMSE diagnosis of CI, participants were divided into 3 categories (Figure 1). According to the results of variance analysis and radar plot (Table 3; Figure 2), except for the STi (*p* = 0.607), the other CI-related indicators in BFSM differed in relation to different clusters (*p* < 0.05). Cluster 1 had higher levels of introspective intensity index (IIi, 63.4 ± 20.0), anxiety tendency index (ATi, 67.2 ± 13.6), emotional resistance index (ERi, 50.2 ± 11.9), hypoxia index (Hi, 41.8 ± 8.3), and lower cognitive efficiency index (CEi); cluster 2 had higher levels of implicit cognitive threshold index (ICTi, 87.2 ± 12.7), CEi (213.8 ± 72.0), and lower ERi, ATi, and STi; cluster 3 had higher levels of STi, and lower levels of CEi, Hi, and IIi. Cluster 1 participants were characterized by anxiety tendency, Cluster 2 participants had a better functional state, and Cluster 3 with reduced brain functional reserve. The similarity between different clusters was assessed using a one-way analysis of similarity (ANOSIM) with a *p* < 0.005 significance level. ANOSIM demonstrated clearly different CI-related indicators between CI Clusters identified by MoCA (R = 0.457, *p* = 0.001) and MMSE (R = 0.442, *p* = 0.001) (Figure 2).

## Discussion

The brain functional state of participants was quantified using BFSM, and they were diagnosed with CI according to MoCA and MMSE scores, respectively. The functional brain indicators associated with CI were strictly selected by LASSO regression analysis and regression analysis. Selected indicators were then used to classify participants with possible CI into five groups (MoCA) and three groups (MMSE). The classification showed good intra-group consistency and inter-group variability and provided a clinical basis for further assessment of each type of CI, which could also be used to guide individualized early rehabilitation programs.

The present study showed that regardless if MoCA and MMSE were used as screening tools for CI, the brain functional state indicator STi was associated with the identification of CI. STi is a composite reflection of the brain’s ability to automatically collect and process external information through sensory organs in brain waves, which reflects the excitability of the sensory nerve center. The decline of STi mainly manifests as a decrease in the attention and sensitivity of the brain to external information. Cognitive neuroscience theory suggests that the human brain engages in information processing, which usually includes different stages such as sensory recognition, central process, and execution (Verwey et al., 2015; Melnik et al., 2017). These stages occur sequentially in the temporal dimension, and sensory recognition is the prerequisites for cognitive processing (Blom et al., 2021). Therefore, the decline of external focus ability may be a risk factor for CI.

Decreased sensory function in older people may be associated with alterations in peripheral sensory organs and sensory cortex, including olfactory dysfunction, age-related hearing impairment (ARHI), and visual impairment; however, the underlying neural architecture and information integration processes are unknown (Misselhorn et al., 2020). Olfactory dysfunction has been defined as a clinical marker for the early diagnosis of various neurodegenerative pathologies, which usually precedes motor and cognitive function symptoms and is mostly seen in the early stages of most Parkinson’s and Alzheimer’s patients (Dan et al., 2021). It was also reported that olfactory dysfunction is correlated with confrontational naming and dysfunction in memory recognition in patients with cognitive dysfunction, where pathological mechanisms may be related to cortical atrophy and white matter abnormalities (Yoo et al., 2018). In addition, ARHI and visual impairment were identified as risk factors for developing CI (Quaranta et al., 2014; Lim et al., 2020).

Based on the predictors screened by the LASSO model, 81 participants diagnosed with cognitive dysfunction by MoCA were classified into 5 clusters. Participants in cluster 1 had lower MRPi, STi, EAi, and MFi, which indicated that participants in this cluster had a better brain thinking ability and did not show excessive tension and uneasiness about the test task. However, since the sensory threshold of cluster 1 participants was lower compared to other clusters, it indicated that the sensory organs of participants in this cluster could be less capable of collecting and processing external information, thus suggesting that such individuals may have some degree of dysfunction in visual and auditory senses. Clinicians need to perform further sensory function assessment of the participants, clarify the type of sensory dysfunction, and timely carry out targeted rehabilitation training to prevent further decline of the cognitive processes in older people due to sensory information input impairment. Reaction speed is a composite reflection of the speed of brain feedback processing information in brain waves.

Cluster 2 participants had faster response speed and lower MRPi and MFi, which showed that the brain function state of these participants was better than that of other clusters. Cluster 3 and cluster 4 participants responded slowly, which may be due to the slow excitatory conduction between cortical and subcortical neurons and the weak ability to transmit and process information. This age-related slowing of sensorimotor abilities may be associated with decreased neuronal recruitment efficiency in older adults (Marusic et al., 2022). In daily life, older people are less likely to timely react to complex environments or obstacles, which may increase their risk of falls. These participants can be combined with physical and cognitive training, which has been demnostrated to improve global cognition and enhance postural performance in senior citizens (Bamidis et al., 2015; Laatar et al., 2018). Cortical network analysis revealed that the combined training scheme could induce neuroplasticity by increasing local information processing or information flow of cortical networks, resulting in better functional organization of brain networks (Zilidou et al., 2018). Longer duration of cognitive activity and increased cognitive load put a greater load on information processing ability. When there is insufficient capacity, individuals experience increased confusion and fatigue in thinking, decreased task completion, and increased error rates in performing tasks. For instance, the brain function tests showed increased MRPi, MFi, and EAi, in the participants in cluster 5. In their study, Wascher found that older people were more likely to experience thought confusion and fatigue than younger when performing prolonged cognitive tasks, which may be caused by reduced efficiency of executive control in the frontal lobes (Wascher and Getzmann, 2014).

Based on the predictors screened by the LASSO model, 33 participants diagnosed with cognitive dysfunction by MMSE were classified into 3 categories. Those in cluster 1 had higher Hi, IIi, ATi, and ERi, and lower CEi. Hi reflects the cortical and subcortical EEG signal complexity changes due to decreased cerebral oxygen saturation. The regulation of cerebral oxygen metabolism is essential for maintaining normal cognitive function, and a correlation has been found between the decrease in local cerebral oxygen saturation levels during the perioperative period and the occurrence of postoperative CI in older patients (Schoen et al., 2011). As Cluster 1 participants have higher Hi, a physician may try to increase the cerebral blood oxygen supply through continuous low-flow oxygen therapy to improve brain functional state. IIi reflects the degree of dynamic dispersion of cortical and subcortical EEG signals. An increase in the IIi manifests as a continuous excitation of various cortical regions, a decrease in coordination, and an increase in the intensity of internal brain thinking that is not under rational control, further exacerbating the degree of brain thinking fatigue. The increase in ERi and ATi may be explained by the fact that during EEG signal acquisition, the participant is not adapted to the environment when faced with maintaining focus under voice prompts, and there is resistance and decreased cooperation with the test. On the other hand, negative emotions may be explained by the poor psychological state of participants, manifesting as irritability, loss of interest, and diminished energy in cognitive activities. Studies have found that depression and anxiety are risk factors for mild CI and Alzheimer’s disease (Li et al., 2016; van Harten et al., 2018). Physicians could use a self-rating anxiety scale (SAS) to further assess the mental state of participants in this cluster and take timely targeted interventions (Perin et al., 2022). For patients with depression, rTMS is an evidence-based treatment that targets the left dorsolateral prefrontal cortex at a high frequency (Mutz et al., 2019). Intermittent theta burst stimulation (iTBS) is a newer form of rTMS which has been shown to be non-inferior to standard 10 Hz rTMS in the reduction of depressive symptoms (Blumberger et al., 2018).

Cluster 2 participants had higher ICTi and CEi, which manifested as cortical inhibition and predominantly high amplitude fast waves. Their brain function was decreased, they showed reduced ability to process information and perform tasks, and they had lower energy expenditure. It was previously reported that the hypometabolic state of the parietal and occipital lobes of the cortex is a predictor of early dementia conversion in Parkinson’s (Baba et al., 2017). Further screening for Parkinson’s and dementia risk should be promptly performed in this group of participants to avoid a progressive decline of function. Cluster 2 participants also had lower STi and required prompt screening for perceptual function. In addition, participants in this cluster had the lowest ERi and ATi, indicating that their emotional state was better than others. Cluster 3 had a higher sensory threshold and lower CEi, Hi, and IIi, and the participants in this cluster had better perceptual and functional brain state.

The study categorized participants according to their brain functional state, further clarifying the characteristics of cognitive decline across clusters. Depending on their brain function, clinicians could use active treatments such as oxygen inhalation, reaction time and visual–auditory training and psychological interventions. These early intervention methods can reduce the negative impact of cognitive decline on patients and slow the progression of cognitive impairment.

In this study, the screening tool MoCA screened a higher number of patients with cognitive impairment. A possible explanation for this might be that the participants in this study were community-dwelling older adults who may have normal cognitive function or have mild declines that are not enough to cause significant limitations in activities of daily living. Previous studies have shown that MoCA is superior to MMSE in distinguishing between mild cognitive impairment and normal individuals (Pinto et al., 2019; Jia et al., 2021). Our finding is consistent with those of previous authors.

### Limitations

One limitation of the present study was the selection of cut-off points of the screening tools. It is desirable to define the cut-off point based on the level of formal education of the older adults. This will more accurately identify seniors at risk for cognitive decline and early onset dementia. No CI participants were not included in this study, and in a follow-up study, we will compare differences in brain function state between no CI participants and different CI clusters. Third, the participants in this study were mainly from the Fall Clinic. The sample size was relatively small, and the range of MoCA and MMSE scores were relatively large. The next step in this study will be to enroll more older adults in the community to improve CI classification based on brain function.

In conclusion, our findings confirmed that multiple indicators in assessing brain functional state are associated with CI in older adults. The characteristics of CI classification included sensory disorders, low reaction speed, reduced brain functional reserve, anxiety or multifunctional disorders. In this study, participants with CI were further classified according to their brain function state, which can help clinicians formulate personalized early intervention methods to slow down the occurrence of dementia.

## Data availability statement

The original contributions presented in the study are included in the article/Supplementary material, further inquiries can be directed to the corresponding authors.

## Ethics statement

The studies involving humans were approved by the Ethics Committee of the Chinese PLA General Hospital (S2022-475). The studies were conducted in accordance with the local legislation and institutional requirements. The participants provided their written informed consent to participate in this study.

## Author contributions

YL, GZ, and MZ participated in the design of this study, and they both performed the statistical analysis and manuscript preparation. GZ and MZ performed the statistical analysis and manuscript preparation. YL, LH, and NaW collected important background information and drafted the manuscript. XS, JZ, and TZ carried out the literature search, data acquisition, and data analysis. YG and NiW participated in the data acquisition. All authors contributed to the article and approved the submitted version.

## Figures and Tables

**Table:** 

ANOSIM	analysis of similarities
ANOVA	analysis of variance
ATi	anxiety tendency index
BFSM	brain functional state measurement
BMRi	brain metabolism rate index
CEi	cognitive efficiency index
CI	cognitive impairment
DTi	depression tendency index
EAi	external apprehension index
EEG	electroencephalogram
ERi	emotional resistance index
Hi	hypoxia index
ICTi	implicit cognitive threshold index
IIi	introspective intensity index
LRM	logistic regression models
MMSE	mini-mental state exam
MoCA	Montreal cognitive assessment
MFi	mental fatigue index
MRPi	mental refractory period index
MTSi	mental task-switching index
STi	sensory threshold index
