# Supplementary material for: Classification of cognitive impairment in older adults based on brain functional state measurement data via hierarchical clustering analysis
Source: Front Aging Neurosci. 2023 Dec 15;15:1198481. doi: 10.3389/fnagi.2023.1198481 (PMC10757366; doi:10.3389/fnagi.2023.1198481)
Supplement: Supplementary file 1 [file Data_Sheet_1.docx]

Supplementary Appendix

This appendix has been provided by the authors to give readers additional information about their work.

**EEG signals analysis by wavelet calculation**

EEG signals consist of both cortical and subcortical components, and during acquisition, electrodes are inevitably affected by eye movements. Since the signal intensity of eye movement is much higher than that of EEG signals, brain waves can be distinguished based on signal intensity. Using wavelet calculation, EEG signals recorded by scalp electrodes can be decomposed into cortical and subcortical components, enabling the identification of weak and high-frequency signals while removing pseudo-difference signals. The purpose of Brain Functional State Monitoring (BFSM) is to reflect the current functional state of the brain, without requiring precise lesion localization. Therefore, EEG signals processed using wavelet calculation may reflect the functional state of different brain regions. BFSM includes 20 indicators.

During the EEG data collection process, the sampling frequency was 1600 Hz, the precision was 16 bits, and the EEG calculation time window was 1 second. After resting for 2 minutes, the patient underwent a 6-minute EEG recording that included eye-closing, eye-opening, and concentration tasks under real-time voice prompts. The wavelet-based EEG analysis software package was used to calculate the collected EEG signals, and the core mechanism of the software was a two-compartment model of brain waves. Specifically, the EEG signal of a certain lead was decomposed into two parts, representing the brain waves of the cortical and subcortical compartments, respectively. Wavelet calculation was then used to determine the dynamic relationship between the two groups of brain waves, extract similar components of cerebral cortex output potential signals, such as reproducible regular changes observed in patients with cognitive dysfunction, anxiety, or depression, and obtain characteristic indicators for quantitatively expressing changes in the functional status of the brain.

**Definition of each indicator**

1. Brain Metabolism Rate index (BMRi)

Brain Metabolism Rate index (BMRi) reflects changes in brain energy expenditure resulting from the excitation of cortical and subcortical areas.

2. Mental Refractory Period index (MRPi)

Mental Refractory Period index (MRPi) reflects the characteristics of subcortical electroencephalogram (EEG) during synchronous elevation of cortical and subcortical signals. Abnormally increased MRPi values indicate excitation of multiple areas of the brain, which can lead to reduced ability to process and discriminate external information.

3. Cognitive Efficiency index (CEi)

Cognitive Efficiency index (CEi) reflects the characteristics of cortical EEG signals during elevated cortical and subcortical signal synchronization. An increase in the CEi index indicates cortical inhibition, which is dominated by high amplitude fast waves and corresponds to decreased brain function.

4. Hypoxia index (Hi)

Hypoxia index (Hi) reflects the decrease in complexity of cortical and subcortical EEG signals caused by decreased cerebral oxygen saturation.

5. Sleep Breathing index (SBi)

Sleep Breathing index (SBi) reflects the degree of decrease in complexity of cortical and subcortical EEG signals due to sleep breathing problems, specifically sleep apnea hypopnea syndrome, induced by brain hypoxia during rest.

6. Emotional Resistance index (ERi)

Emotional Resistance index (ERi) reflects the degree of dysregulation of cortical and subcortical information pathways caused by reduced coordination between cortical centers and increased communication with subcortical information pathways. A higher ERi suggests impaired integration of subjective consciousness with the environment.

7. Anxiety Tendency index (ATi)

8. Depression Tendency index (DTi)

Anxiety Tendency index (ATi) and Depression Tendency index (DTi) reflect the degree of coordination and complexity of cortical and subcortical EEG signals.

9. External Apprehension index (EAi)

External Apprehension index (EAi) reflects the degree of β-band dispersion in the cerebral cortex.

10. Sleeping index (Si)

Sleeping index (Si) reflects the rate at which the density of cortical-subcortical information interaction decreases during sleep. An increase in the index indicates a longer period of cortical and subcortical EEG signals maintaining a certain level of complexity before decreasing.

11. Selective Attention index (SAi)

Selective Attention index (SAi) reflects the degree of passive opening of the sensory-perceptual (peripheral nerve) pathways during attentional processes.

12. Mental Inhibition index (MIi)

Mental Inhibition index (MIi) reflects the degree of subcortical inhibition of the cortex.

13. Mental Resilience index (MRi)

Mental Resilience index (MRi) reflects the degree of continuity of neural pathways and information transmission sensitivity to changes in neural task pathways in the brain. An increase in MRi indicates slower diffusion of EEG signal changes in the cortical and subcortical functional areas.

14. Brain Coordination index (BCi)

Brain Coordination index (BCi) measures the appearance of non-periodic, elevated complexity, and real-variable characteristic performance of EEG signals between cortical areas of the brain. An increase in BCi indicates higher complexity and coordination of EEG signals between cortical areas.

15. Working Memory Function index (WMFi)

Working Memory Function index (WMFi) reflects the degree of consolidation of past cognitive pathways in the brain, and is a measure of the degree of specific static synchronization within the cerebral cortex.

16. Sensory Threshold index (STi)

Sensory Threshold index (STi) reflects the degree of excitation of sensory nerve centers. A decrease in STi indicates that the sensory nerve centers are predominantly inhibited while the other centers may be excited.

17. Introspective Intensity index (IIi)

Introspective Intensity index (IIi) is a measure of the degree of coordination between the cerebral cortex and subcortical EEG signals, with a higher IIi reflecting a greater degree of dysfunction and decreased coordination between different regions of the cortex.

18. Implicit Cognitive Threshold index (ICTi)

Implicit Cognitive Threshold index (ICTi) reflects the density of independent neural pathways in the brain, and an increase in this index indicates a better ability to engage in perceptual information processing and perform cognitive tasks.

19. Mental Fatigue index (MFi)

Mental Fatigue index (MFi) reflects the degree of reduction in information exchange between the left and right hemispheres of the brain. An increase in MFi is manifested by sustained hyperexcitability of the right cerebral cortex mapping to the right subcortical areas, which can lead to a decrease in the sensitivity of information transmission pathways and may result in reduced cognitive performance and increased fatigue.

20. Mental Task-Switching index (MTSi)

Mental Task-Switching index (MTSi) reflects the ability of the brain to flexibly switch between different mental tasks by measuring changes in nerve conduction priorities within cortical and subcortical neuronal cells.

| **TableS**  Table S1 Basic information of the cluster participants diagnosed by MoCA   \| Name \| Levels \| No CI  (N=53) \| Cluster 1 (N=21) \| Cluster 2  (N=18) \| Cluster 3  (N=8) \| Cluster 4  (N=22) \| Cluster 5  (N=12) \| p \| \| --- \| --- \| --- \| --- \| --- \| --- \| --- \| --- \| --- \| \| Sex \| Male \| 18 (34%) \| 11 (52.4%) \| 7 (38.9%) \| 6 (75%) \| 16 (72.7%) \| 6 (50%) \| .026 \| \|  \| Female \| 35 (66%) \| 10 (47.6%) \| 11 (61.1%) \| 2 (25%) \| 6 (27.3%) \| 6 (50%) \|  \| \| Age \| Mean ± SD \| 68.7 ± 6.9 \| 73.3 ± 10.1 \| 72.7 ± 8.2 \| 78.0 ± 12.6 \| 82.8 ± 10.2 \| 84.4 ± 8.3 \| <.001 \| \| Education \| Middle school \| 6 (11.3%) \| 5 (23.8%) \| 3 (16.7%) \| 2 (25%) \| 2 (9.1%) \| 2 (16.7%) \| .735 \| \|  \| College \| 22 (41.5%) \| 7 (33.3%) \| 10 (55.6%) \| 2 (25%) \| 8 (36.4%) \| 6 (50%) \|  \| \|  \| University \| 25 (47.2%) \| 9 (42.9%) \| 5 (27.8%) \| 4 (50%) \| 12 (54.5%) \| 4 (33.3%) \|  \| \| Visuospatial.executive \| Mean ± SD \| 4.5 ± 0.6 \| 3.2 ± 1.3 \| 3.4 ± 1.2 \| 3.4 ± 0.9 \| 2.8 ± 1.3 \| 2.8 ± 1.0 \| <.001 \| \| Naming \| Mean ± SD \| 2.9 ± 0.3 \| 2.8 ± 0.4 \| 2.7 ± 0.8 \| 3.0 ± 0.0 \| 2.5 ± 0.9 \| 2.5 ± 0.7 \| .056 \| \| Attention \| Mean ± SD \| 5.8 ± 0.4 \| 5.3 ± 0.8 \| 5.0 ± 1.2 \| 5.5 ± 0.5 \| 5.0 ± 1.4 \| 5.2 ± 0.9 \| <.001 \| \| Language \| Mean ± SD \| 2.2 ± 0.7 \| 1.8 ± 0.7 \| 1.8 ± 0.7 \| 2.0 ± 0.5 \| 1.8 ± 1.1 \| 1.8 ± 1.1 \| .128 \| \| Abstraction \| Mean ± SD \| 1.9 ± 0.4 \| 1.4 ± 0.7 \| 1.1 ± 0.7 \| 1.6 ± 0.5 \| 1.2 ± 0.8 \| 1.6 ± 0.7 \| <.001 \| \| Delayed.recall \| Mean ± SD \| 3.9 ± 0.8 \| 1.8 ± 1.6 \| 2.1 ± 1.9 \| 1.8 ± 1.0 \| 1.5 ± 1.6 \| 1.0 ± 1.5 \| <.001 \| \| Orientation \| Mean ± SD \| 5.9 ± 0.2 \| 5.6 ± 1.0 \| 5.3 ± 1.4 \| 5.6 ± 0.5 \| 5.3 ± 1.2 \| 5.3 ± 1.2 \| .034 \| \| MoCA \| Mean ± SD \| 27.3 ± 1.3 \| 21.9 ± 3.3 \| 21.7 ± 4.8 \| 23.0 ± 1.2 \| 20.3 ± 4.8 \| 20.3 ± 4.4 \| <.001 \|   Table S2 Basic information of the cluster participants diagnosed by MMSE   \| Name \| Levels \| No CI  (N=101) \| Cluster 1  (N=5) \| Cluster 2  (N=12) \| Cluster 3  (N=16) \| p \| \| --- \| --- \| --- \| --- \| --- \| --- \| --- \| \| Sex \| Male \| 44 (43.6%) \| 3 (60%) \| 7 (58.3%) \| 10 (62.5%) \| .400 \| \|  \| Female \| 57 (56.4%) \| 2 (40%) \| 5 (41.7%) \| 6 (37.5%) \|  \| \| Age \| Mean ± SD \| 72.3 ± 9.4 \| 82.0 ± 13.8 \| 79.4 ± 9.2 \| 79.8 ± 12.8 \| .003 \| \| Education \| Middle school \| 14 (13.9%) \| 0 (0%) \| 2 (16.7%) \| 4 (25%) \| .834 \| \|  \| College \| 42 (41.6%) \| 3 (60%) \| 5 (41.7%) \| 5 (31.2%) \|  \| \|  \| University \| 45 (44.6%) \| 2 (40%) \| 5 (41.7%) \| 7 (43.8%) \|  \| \| Orientation.1 \| Mean ± SD \| 9.9 ± 0.4 \| 7.4 ± 3.8 \| 7.6 ± 3.7 \| 9.5 ± 1.1 \| <.001 \| \| Registration \| Mean ± SD \| 3.0 ± 0.2 \| 2.4 ± 1.3 \| 2.5 ± 1.0 \| 2.9 ± 0.2 \| <.001 \| \| Attention.and.Calculation \| Mean ± SD \| 4.8 ± 0.5 \| 2.6 ± 1.8 \| 2.8 ± 1.6 \| 3.6 ± 1.4 \| <.001 \| \| Recall \| Mean ± SD \| 2.1 ± 1.0 \| 0.2 ± 0.4 \| 0.6 ± 0.8 \| 0.6 ± 0.7 \| <.001 \| \| Language.and.Praxis \| Mean ± SD \| 8.9 ± 0.4 \| 8.2 ± 1.1 \| 7.8 ± 1.5 \| 8.6 ± 0.7 \| <.001 \| \| MMSE \| Mean ± SD \| 28.5 ± 1.2 \| 20.8 ± 4.3 \| 21.2 ± 6.2 \| 25.2 ± 1.1 \| <.001 \| |
| --- | --- | --- | --- | --- | --- | --- | --- | --- | --- | --- | --- | --- | --- | --- | --- | --- | --- | --- | --- | --- | --- | --- | --- | --- | --- | --- | --- | --- | --- | --- | --- | --- | --- | --- | --- | --- | --- | --- | --- | --- | --- | --- | --- | --- | --- | --- | --- | --- | --- | --- | --- | --- | --- | --- | --- | --- | --- | --- | --- | --- | --- | --- | --- | --- | --- | --- | --- | --- | --- | --- | --- | --- | --- | --- | --- | --- | --- | --- | --- | --- | --- | --- | --- | --- | --- | --- | --- | --- | --- | --- | --- | --- | --- | --- | --- | --- | --- | --- | --- | --- | --- | --- | --- | --- | --- | --- | --- | --- | --- | --- | --- | --- | --- | --- | --- | --- | --- | --- | --- | --- | --- | --- | --- | --- | --- | --- | --- | --- | --- | --- | --- | --- | --- | --- | --- | --- | --- | --- | --- | --- | --- | --- | --- | --- | --- | --- | --- | --- | --- | --- | --- | --- | --- | --- | --- | --- | --- | --- | --- | --- | --- | --- | --- | --- | --- | --- | --- | --- | --- | --- | --- | --- | --- | --- | --- | --- | --- | --- | --- | --- | --- | --- | --- | --- | --- | --- | --- | --- | --- | --- | --- | --- | --- | --- | --- | --- | --- | --- | --- | --- | --- | --- | --- | --- | --- | --- | --- | --- | --- | --- | --- | --- | --- | --- | --- | --- | --- | --- | --- | --- | --- | --- | --- | --- | --- | --- |

**FigureS1**


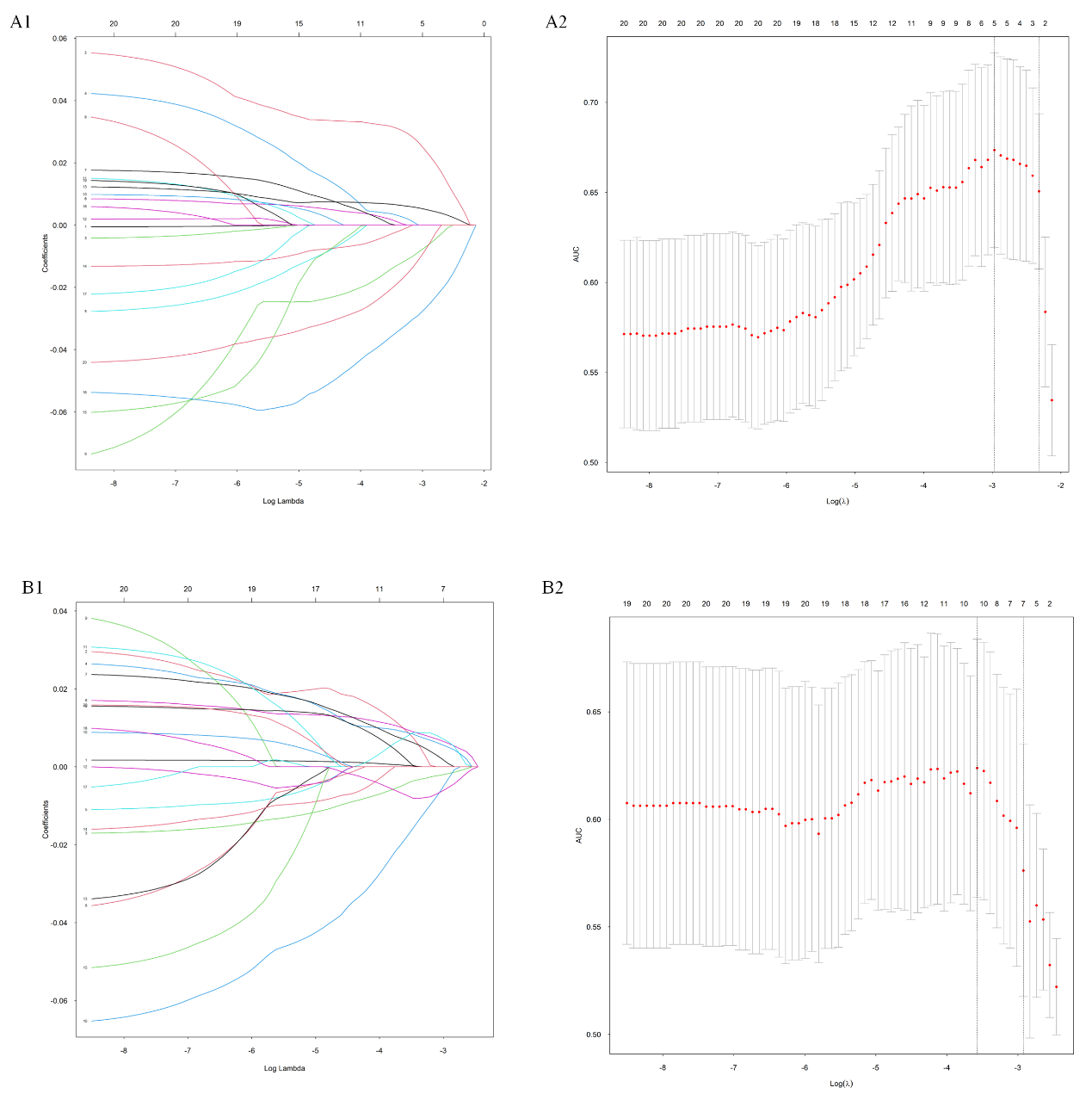


Figure S1 LASSO regression model for predictors of CI diagnosed by (A) MoCA and (B) MMSE.

A1: The relationship between parameter lambda and regression coefficient in LASSO regression; A2: The variation curve of variable coefficient with lambda logarithm in LASSO regression; B1: The relationship between parameter lambda and regression coefficient in LASSO regression; B2: The variation curve of variable coefficient with lambda logarithm in LASSO regression.

**FigureS2**


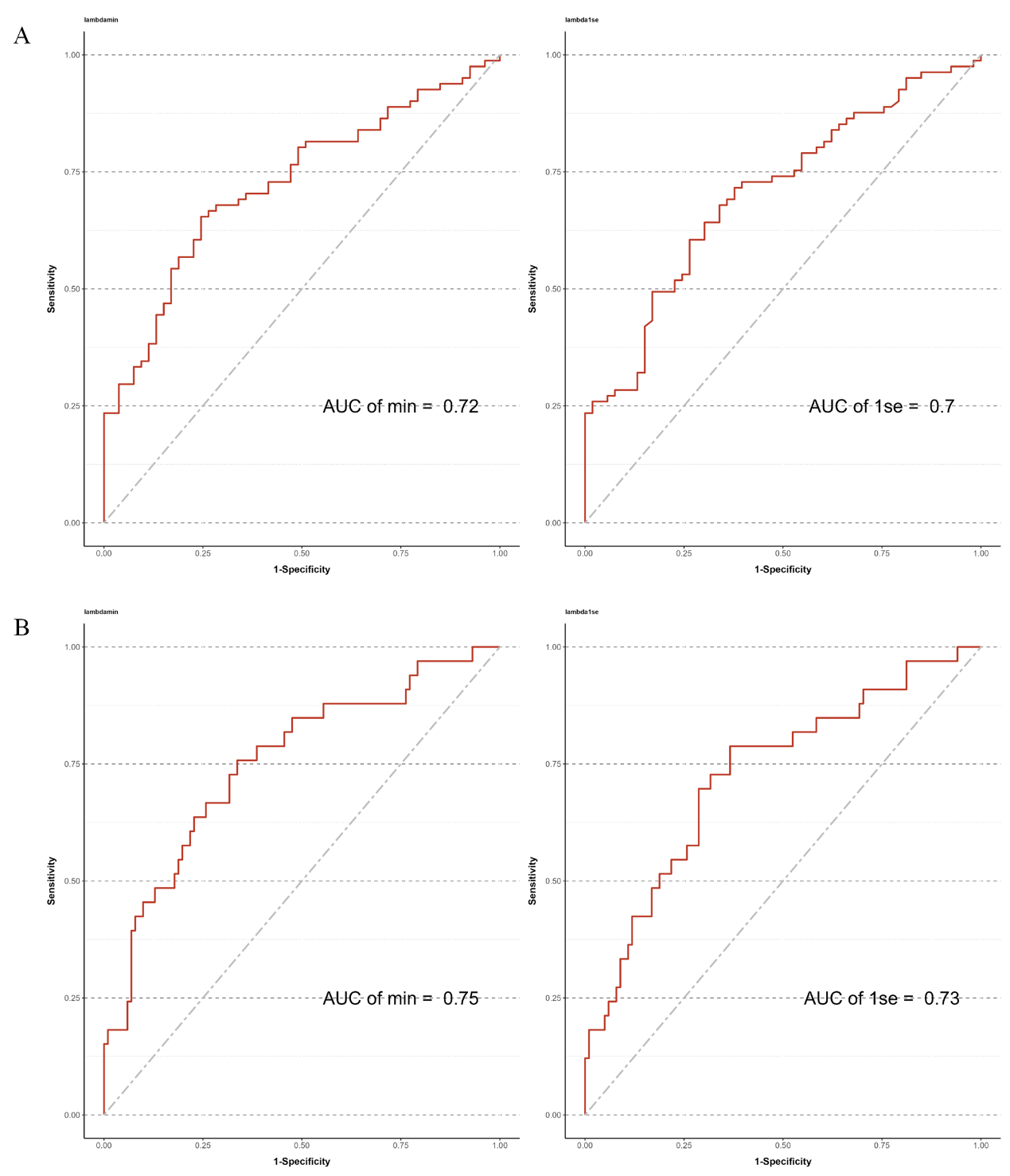


FigureS2 The AUC of the two Lasso regression model (A)MoCA and (B)MMSE.

**FigureS3**


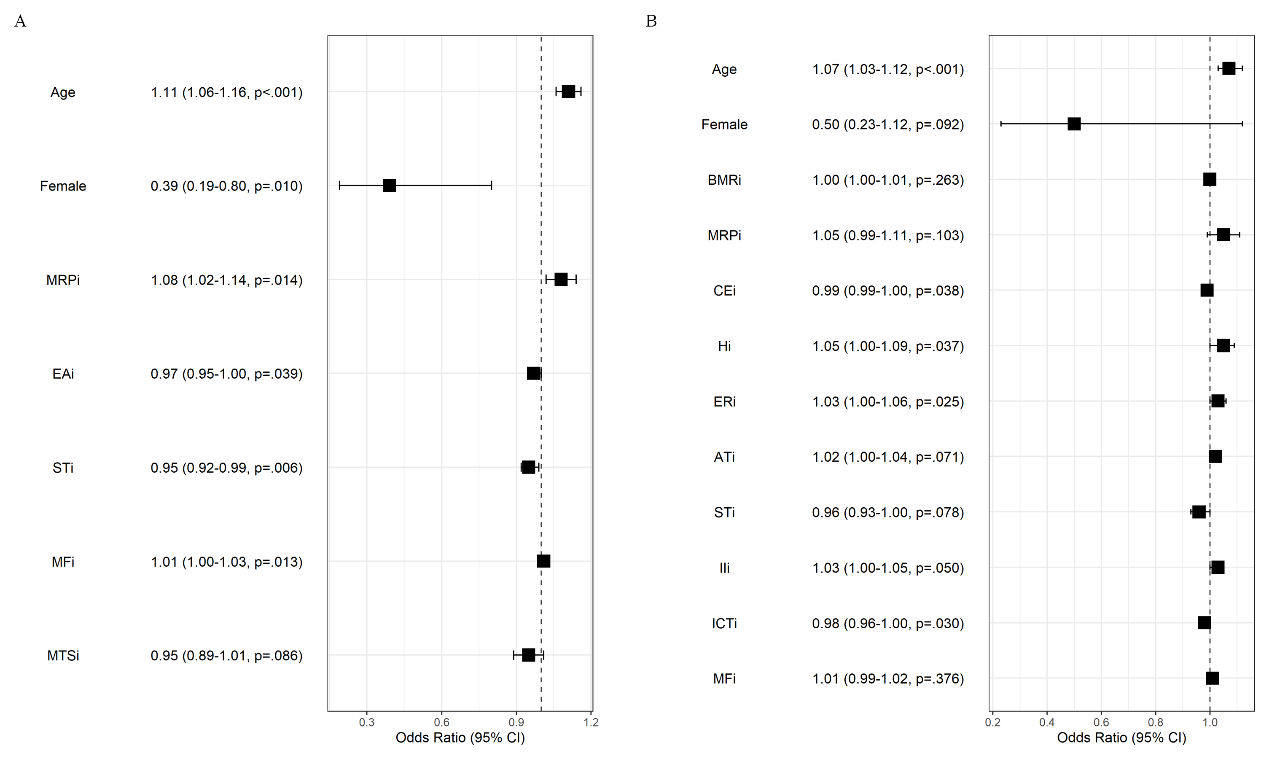


Figure S3 Univariate regression of the screened variables of LASSO regression.

(A) The LASSO regression model with MoCA as the diagnostic criterion for CI.

(B) The LASSO regression model with MMSE as the diagnostic criterion for CI.

**FigureS4**


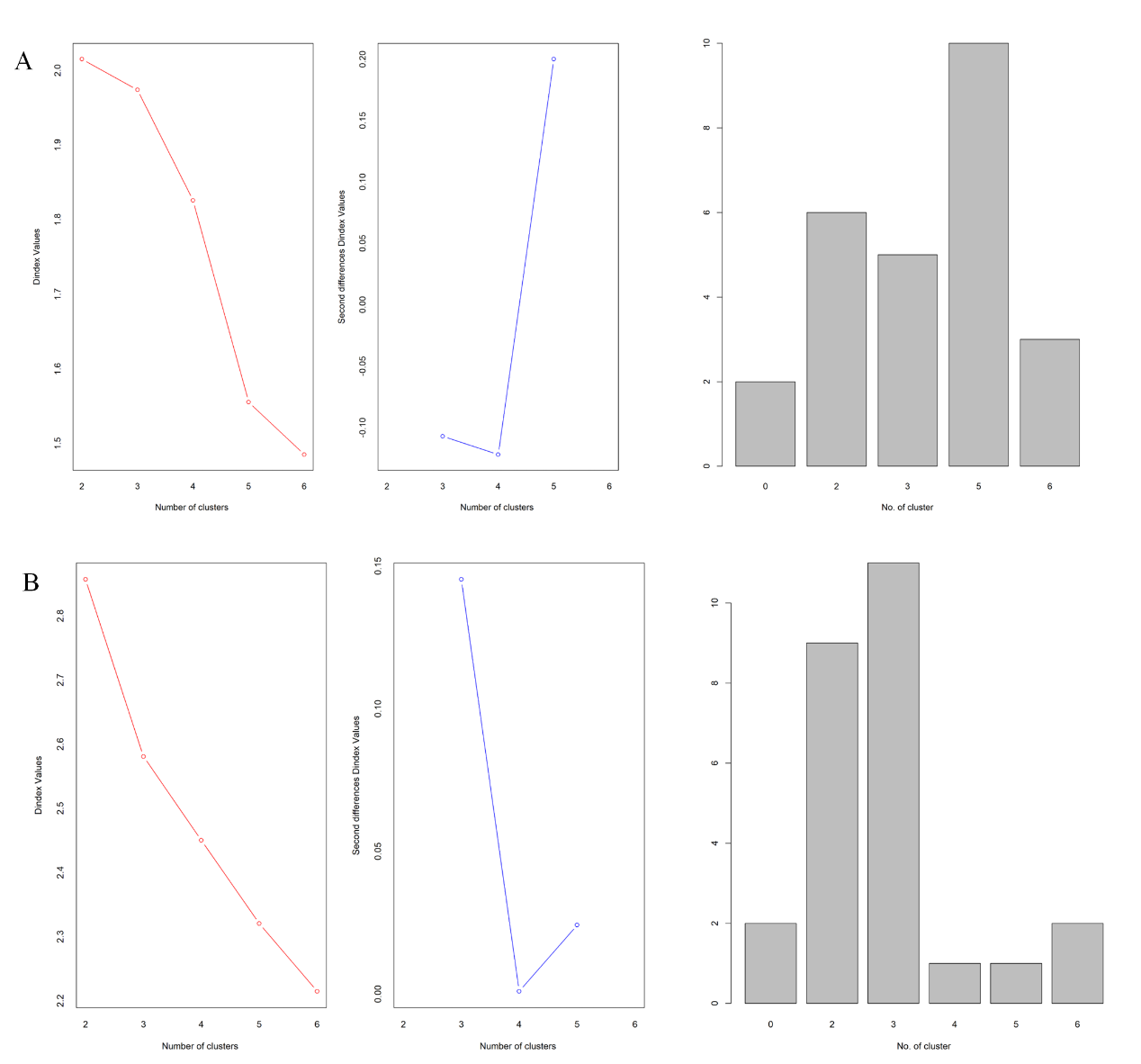


FigureS4 Voting method to determine the optimum number of clusters.

(A) Participants with CI diagnosed by MoCA.

(B) Participants with CI diagnosed by MMSE.
